# Genome evolution through polyploidy: Enhancing plant stress resilience in agriculture

**DOI:** 10.1073/pnas.2522064123

**Published:** 2026-05-26

**Authors:** Patrick P. Edger, Melanie J. A. Body, Sonia De Donno, Adrian E. Platts, Jianrong Wang, Jiming Jiang

**Affiliations:** ^a^Department of Horticulture, Michigan State University, East Lansing, MI 48824; ^b^Genetics and Genome Sciences, Michigan State University, East Lansing, MI 48824; ^c^Honors College, Michigan State University, East Lansing, MI 48824; ^d^Department of Computational Mathematics, Science, and Engineering, Michigan State University, East Lansing, MI 48824; ^e^Department of Plant Biology, Michigan State University, East Lansing, MI 48824

**Keywords:** polyploidy, stress resilience, genome evolution, agriculture, whole genome duplication

## Abstract

Polyploidy, also known as whole genome duplication, is a major evolutionary force in plants, driving diversification and the generation of novel phenotypic variation, including superior abiotic and biotic stress tolerance. The enhanced stress resilience observed in certain polyploids is hypothesized to arise from dynamic epigenetic and genetic changes, including variations in gene content and cis-regulatory elements (CREs), that emerge following polyploidization. These changes directly impact various regulatory, signaling, and metabolic pathways associated with stress response and adaptation. Within polyploid populations, processes like gene duplications, fractionation, and homoeologous exchanges actively shape novel gene content variation, while, simultaneously, alterations in CREs (DNA sequences controlling gene expression) lead to diverse regulatory patterns. This dynamic interplay between changes in gene content and regulation further contributes to expanded phenotypic variation, including enhanced stress resilience. We discuss how advanced genomic and epigenomic techniques, such as pangenomics and single-cell assay for transposase-accessible chromatin with sequencing, are used to uncover these variations and outline new bioinformatic approaches to reveal the underlying genetics of stress resilience and adaptation. Finally, we summarize what remains poorly understood to guide future research, with the goal of unlocking the full potential for enhancing resilience in polyploid crops.

Global agricultural productivity faces an unprecedented challenge from the increasing frequency and intensity of various abiotic and biotic stresses (1). Abiotic stressors, such as extreme temperatures, drought, flooding, and salinity, are directly exacerbated by shifting global weather patterns, which severely impacts crop yields worldwide (2). Simultaneously, biotic stresses, including novel pest outbreaks and disease epidemics, are also influenced by changing environmental conditions, further threatening food production (3). This complex interplay of escalating stresses necessitates the urgent development and deployment of superior climate-resilient crops to ensure global food security for a growing population (4).

Polyploidy, defined as the presence of three or more complete sets of chromosomes, is a major evolutionary force in the plant kingdom, providing the genetic foundation for novel phenotypic variation and the evolution of novel traits over deeper time (5). Polyploidy arises specifically from a failure during mitosis or meiosis that results in genome doubling. The two primary categories of polyploidy are autopolyploidy, where extra chromosome sets originate from a single species, or allopolyploidy, which results from the hybridization of two or more distinct species combined with genome doubling (6). Allopolyploids, including many agriculturally important species, face the added genetic complexity of containing distinct diploid progenitor genomes, requiring mechanisms to regulate these combined, sometimes divergent, genetic contributions (7). While autopolyploids and allopolyploids represent two distinct conceptual categories, not all polyploid individuals will cleanly fall into one of these two classifications; instead, a spectrum of origins exists. A polyploid event occurring within a single individual is a clear case of autopolyploidy, whereas hybridization between highly divergent species (not immediate sister species) with different base chromosome numbers, where the initial interspecific hybrid is not viable, would be supportive of an allopolyploid origin following genome doubling. However, many polyploids exist somewhere along this continuum, a complexity that can also be shaped by species descriptions and the extent of parental divergence. For an excellent and comprehensive review of this topic, please refer to ref. 8.

Following a polyploidization event, genomes undergo a gradual process of diploidization (i.e., returning to a diploid-like state), which involves sequence loss (referred to as fractionation) and various chromosomal changes, including fusion events (5, 9). Thus, over deep evolutionary time, these polyploids will no longer possess three or more complete sets of chromosomes, as was evident immediately after polyploidy. However, these older events can be identified using advanced cytological approaches for mesopolyploid events (10), and through comparative genomic analyses for more ancient polyploidization events (11).

Recent polyploidy is particularly prevalent and important in many key crop species, where it often confers advantages such as increased vigor, larger organs, enhanced adaptability to diverse environments, and improved resilience to various stresses. For instance, tetraploid (4×) species like cotton possess four sets of chromosomes, while hexaploid (6×) wheat has six sets, and octoploid (8×) strawberry boasts eight (12). This widespread occurrence and beneficial impact establish polyploidy as a critical mechanism in the development and ongoing improvement of many agriculturally important crops.

A notable advantage observed in some polyploid plants, including both wild species and cultivated crops, is their enhanced resilience to various environmental stresses compared to their diploid counterparts (13). This heightened stress tolerance extends to both abiotic factors, such as drought, extreme temperatures, salinity, and nutrient deficiencies, and biotic challenges, including pests and diseases. The increased stress resilience often observed in polyploids is hypothesized to be driven by a combination of genetic changes, particularly gene content and cis-regulatory variation, and epigenetic changes leading to novel phenotypic diversity (Fig. 1) (14). These genetic changes can either emerge de novo following the polyploidization event and/or represent preexisting differences that evolved among the progenitor species since their most recent common ancestor. It is important to note that allopolyploids are not expected to be merely the genetic average of their progenitor species, owing to the inherently nonlinear nature of gene expression. Indeed, significant and potentially beneficial changes in gene expression can occur immediately as a direct result of the interspecific hybridization event (15, 16). The presence of multiple gene copies following whole-genome duplication provides a rich substrate for evolution, allowing for the retention (i.e., functional redundancy), subfunctionalization (i.e., partitioning of ancestral functions between duplicate genes), or neofunctionalization (i.e., evolving novel function) of genes (17). Simultaneously, changes in cis-regulatory elements (CREs)—DNA sequences that control the spatiotemporal and abundance patterns of gene expression—can lead to diverse and novel patterns of gene activity (Fig. 1*A*) (18). This genetic diversity found in polyploid populations is further modulated by epigenetic changes, such as DNA methylation and histone modifications, which can influence gene expression without altering the underlying DNA sequence (Fig. 1 *B*–*D*) (7). Genetic alterations in both gene content and CREs, along with an additional layer of epigenetic changes, contribute significantly to the expanded phenotypic diversity and enhanced adaptive capabilities that enable polyploids to thrive under challenging environmental conditions (19).

**Fig. 1.**
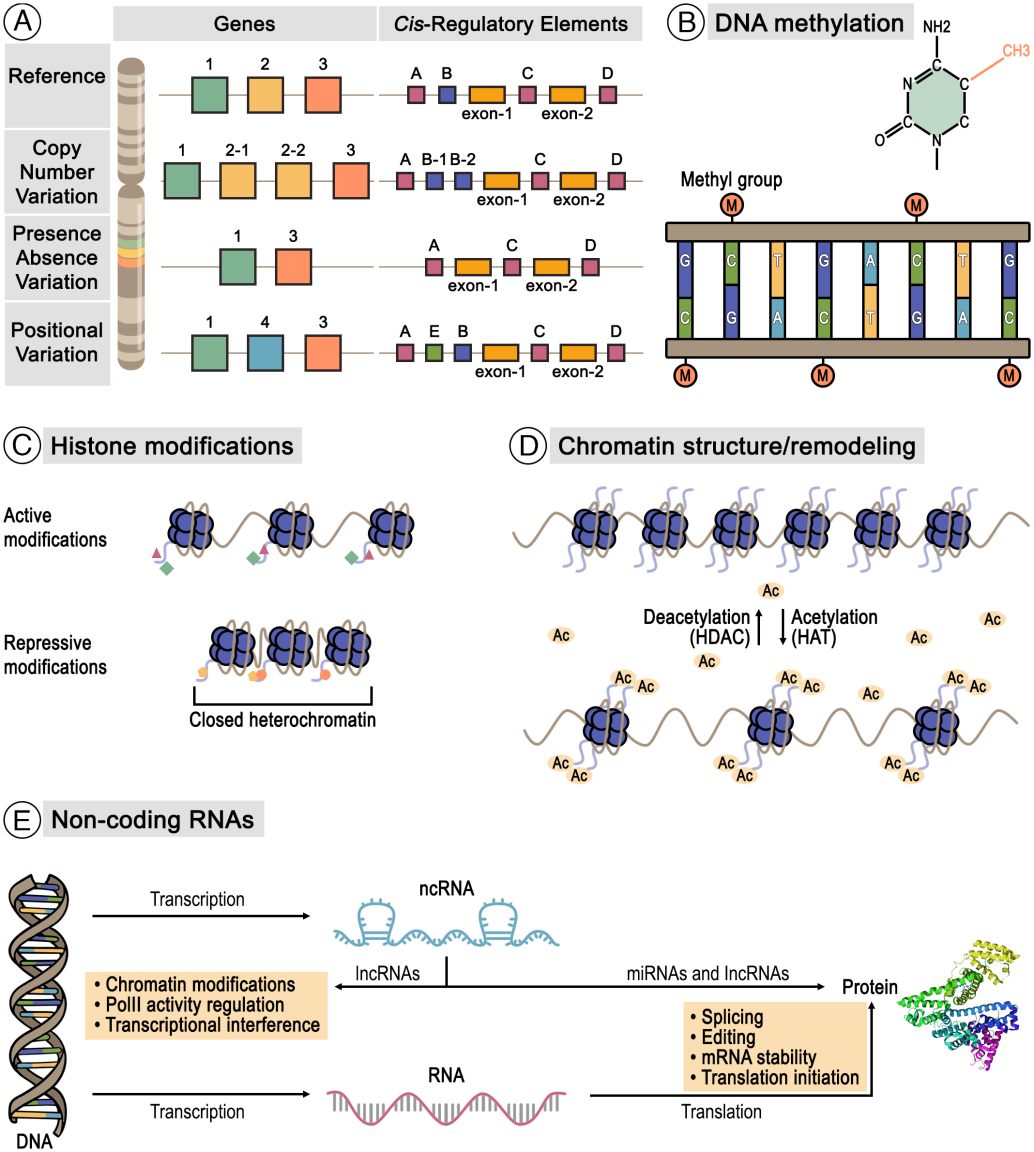
Gene and CRE variation driving phenotypic diversity. Panel (*A*) summarizes various forms of genetic variation that contribute to phenotypic diversity. The left side depicts the gene content variation relative to a reference genome (*Top* row). The second row demonstrates CNV, specifically the duplication of Gene #2, resulting in two copies compared to the reference. The third row illustrates PAV, where Gene #2 is entirely absent from the genomic region compared to the reference. The *Bottom* row highlights Positional Variation of a gene, showing Gene #4 having transposed from another genomic location into this region. The *Right* panel depicts CRE Variation associated with a gene. The *Top* row displays the arrangement of CREs in a reference gene that are located upstream (CRE “A” and “B”), intronic (CRE “C”), and downstream (CRE “D”). The second row shows CNV of CRE “B,” with two copies present compared to the reference. The third row illustrates PAV, depicting the complete loss of CRE “B” from the region. The *Bottom* row illustrates Positional Variation of a CRE, where CRE “E” has transposed from another genomic location into this region. Polyploidization can induce TE activity, through the disruption of epigenetic silencing, which in turn increases the rate of CRE transposition arising from TEs (20). Collectively, these various types of genetic and regulatory alterations provide the raw material for phenotypic variation and adaptation. Panel (*B*) depicts DNA methylation (M). DNA methylation is an epigenetic mechanism where a methyl group is added to the DNA molecule, typically at the cytosine bases within CpG dinucleotide sites. This process does not alter the DNA sequence itself but acts as a molecular switch to regulate gene expression, often leading to gene repression or silencing. Panel (*C*) depicts Histone modifications. Histone modifications are epigenetic marks that influence gene expression by changing the structure of chromatin—the complex of DNA and histone proteins that make up chromosomes. These modifications, such as acetylation, methylation, and phosphorylation of histone tails, can lead to either an open, transcriptionally active state (euchromatin) or a condensed, transcriptionally repressive state (closed heterochromatin), which ultimately results in gene silencing. Panel (*D*) depicts chromatin remodeling. Chromatin remodeling involves changes in the structure of the chromatin, the complex of DNA and histone proteins that makes up chromosomes. Histone acetyltransferases (HATs) add acetyl groups to histones, which loosens chromatin and promotes an open structure, making DNA more accessible for gene transcription. Conversely, histone deacetylases (HDACs) remove these acetyl groups, leading to a condensed or closed chromatin state that represses gene expression. Panel (*E*) depicts noncoding RNAs. Noncoding RNAs are RNA molecules that are transcribed from DNA but are not translated into proteins, instead functioning directly to regulate gene expression and other cellular processes. Noncoding RNAs, includes lncRNA and miRNA, modify chromatin, regulate PolIII activity, and interfere with transcription, as well as influencing splicing, editing, mRNA stability, and the initiation of translation.

Here, we aim to review the current knowledge on how genomic changes are generated following polyploidization, which ultimately influences phenotypic variation, including enhanced stress resilience. We outline the current methods and recent technical advances used to study this variation. Finally, we highlight what remains poorly understood and propose specific questions to guide future research, with the goal of unlocking the full potential for enhancing resilience in polyploid crops. Closing these fundamental knowledge gaps is essential for developing predictive models that can guide the strategic selection and engineering of polyploid crops to translate these discoveries into solutions for agriculture.

## Uncovering Gene Content Variation Associated with Stress Resilience

**Fig. 2.**
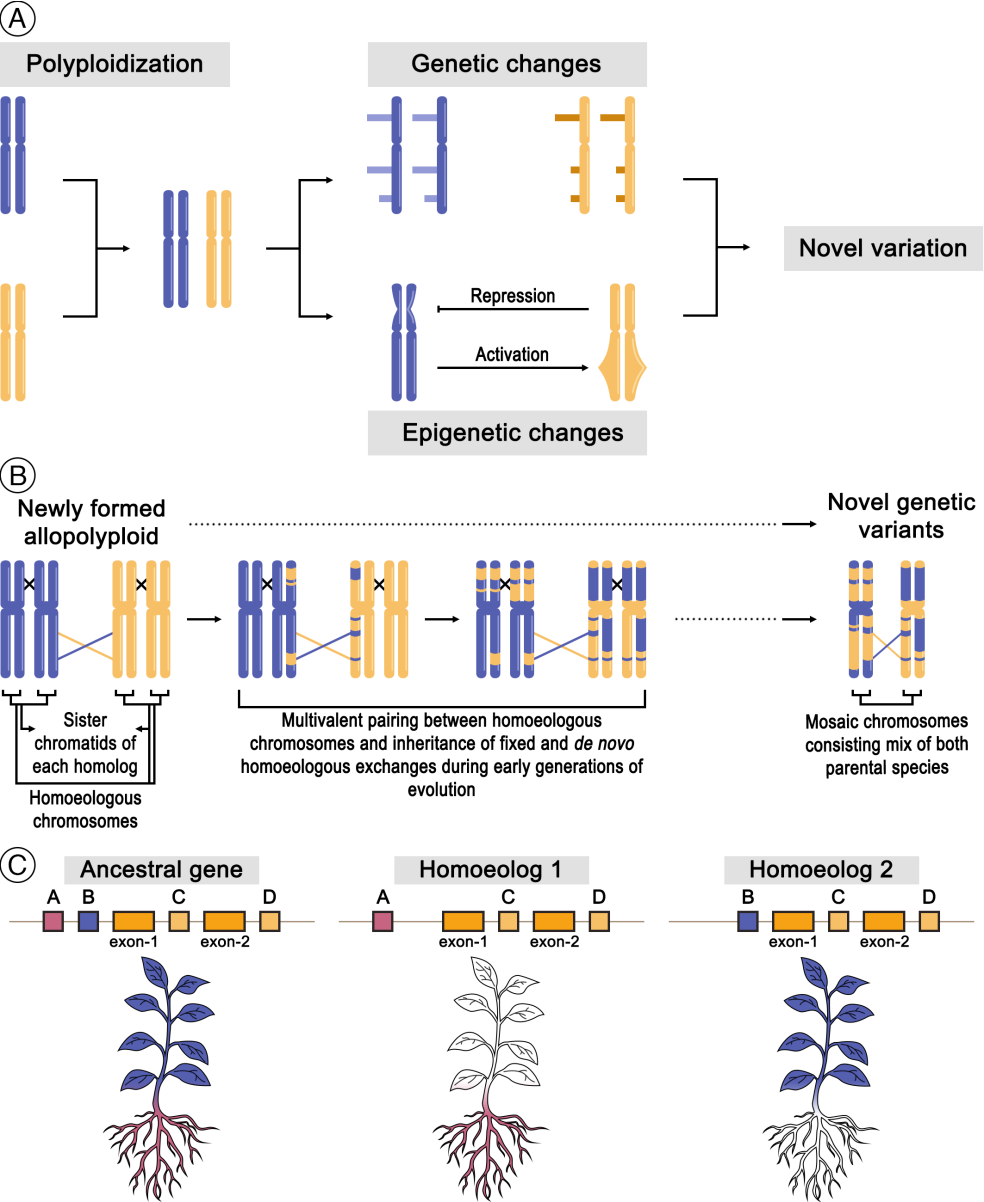
Polyploidy as a driver of genetic diversity and adaptation. This figure illustrates the dynamic genetic and epigenetic changes that occur following polyploidization. Panel (*A*) depicts the initial formation of a new allopolyploid organism through the hybridization and subsequent genome duplication of two distinct diploid progenitor species (represented by blue and yellow chromosomes). Following the event, the newly formed polyploid undergoes a series of genetic and epigenetic changes over time. These cumulative alterations collectively result in the emergence of novel phenotypic variation that was not observed in either of the original diploid progenitor species (adapted from ref. 23). Panel (*B*) depicts how HEs—recombination events between chromosomes from different progenitor genomes—occur over successive generations in newly formed polyploids (adapted from ref. 24). These HEs lead to the creation of novel genetic variants, including altered dosage of parental allele copies (e.g., gain or loss of specific gene copies) as well as the duplication and/or loss of entire genes or gene content within the subgenomes. Panel (*C*) illustrates how the loss of CREs, a known phenomenon among duplicated genes in homoeologs, can drive diverged gene expression patterns (18). For instance, an ancestral gene (expressed in both roots and shoots, containing both CRE “A” and “B”) might, after polyploidization and CRE loss, result in one gene copy being solely expressed in a specific spatial-temporal context (e.g., copy A for roots only) while the other gene copy is expressed solely in the remaining contexts (e.g., copy B for shoots only). In addition to these genetic changes, a series of epigenetic modifications, which are known to occur in newly formed polyploids, may further alter the accessibility of these regulatory regions, contributing to the observed expression divergence.

Gene content variation, which includes the presence, absence, and differing copy numbers of specific genes, is a fundamental source of genetic diversity among individuals within a species (Figs. 1 and 2*A*) (21). This variation profoundly influences gene expression abundance and, ultimately, an organism’s phenotype including its ability to respond to environmental stimuli. For improving stress resilience in crop plants, understanding gene content variation is important, as the gain or loss of particular genes can directly impact metabolic pathways, signaling cascades, and defense mechanisms vital for adapting to challenging abiotic and biotic stressors (22).

Within polyploids, gene content variation arises from several dynamic evolutionary processes. Following a polyploidization event, gene fractionation—the selective loss of duplicated genes—systematically reduces the initial largely redundant gene content (9). In an allopolyploid, some novel gene content is contributed by the different diploid progenitor species, of which one may be inherently more resilient to a particular abiotic stress or resistant to specific pathogens (25). Fractionation can occur asymmetrically between the subgenomes—the constituent genomes inherited from the different diploid ancestors—leading to a differential loss of genes and their associated regulatory elements (26). Additionally, homoeologous exchanges (HEs), which are recombination events between homoeologous chromosomes, contributed by the different diploid progenitors, can alter the precise dosage of parental allele copies for genes (24) (Fig. 2*B*). Beyond these, ongoing tandem and segmental duplications continuously generate new gene copies, further enriching the gene content landscape (27). These diverse forms of gene content variation, driven by both loss and gain, are not static; instead, they actively segregate both between and within polyploid populations, offering a rich substrate for selection and adaptation to diverse environments.

The increased dosage of a gene product can significantly modify the flux within metabolic pathways (27). For example, increasing the dosage of an enzyme that catalyzes a specific reaction can accelerate that step, potentially pulling more substrate through its pathway (28). This can have broader implications if that substrate is also utilized by another enzyme in a different, competing pathway, leading to altered metabolite profiles. Furthermore, polyploidy may alter the cell’s surface area to volume balance, which can itself fundamentally reshape metabolic profiles (29). Similarly, the dosage of a regulatory gene, such as a transcription factor (TF), can profoundly modify the timing or magnitude of a particular trait (30). This is especially true if that regulator is involved in a delicate balance with other activators or repressors, where even subtle dosage imbalances can reconfigure developmental trajectories.

Recent advances in genomics have revolutionized our ability to identify and characterize gene content variation. Comparative genomics, which analyzes and compares the genomes of multiple individuals or closely related species, offers a powerful way to pinpoint regions of presence–absence variation (PAV) and copy number variation (CNV) (31). The development of pangenomics enhances this capability by capturing the entire gene repertoire of a species, moving beyond single reference genomes and toward reference graphs (32). This approach provides a comprehensive catalog of both core genes (shared by all individuals) and accessory genes (present only in some). In many plant pangenomes, a large fraction of the genes (over 50% in some studies) were identified as accessory (e.g., ref. 33). Many of these accessory genes contribute to important adaptive traits, including abiotic stress tolerance and disease resistance (34).

Leveraging various emerging techniques that combine genomic data with experimental and computational approaches, researchers can now systematically identify gene content variation that contributes to improved stress tolerance. For instance, by comparing gene content across diverse accessions exposed to specific abiotic or biotic stresses and integrating this with transcriptomic data, we can pinpoint genes whose presence, absence, or copy number directly correlates with differential gene expression and altered stress tolerance phenotypes (35). These insights can then be combined with genome-wide association studies and/or quantitative trait loci analyses to identify specific genomic regions associated with superior stress tolerance or variation of a particular trait (36).

## Uncovering Cis-Regulatory Regions Associated with Stress Resilience

**Fig. 3.**
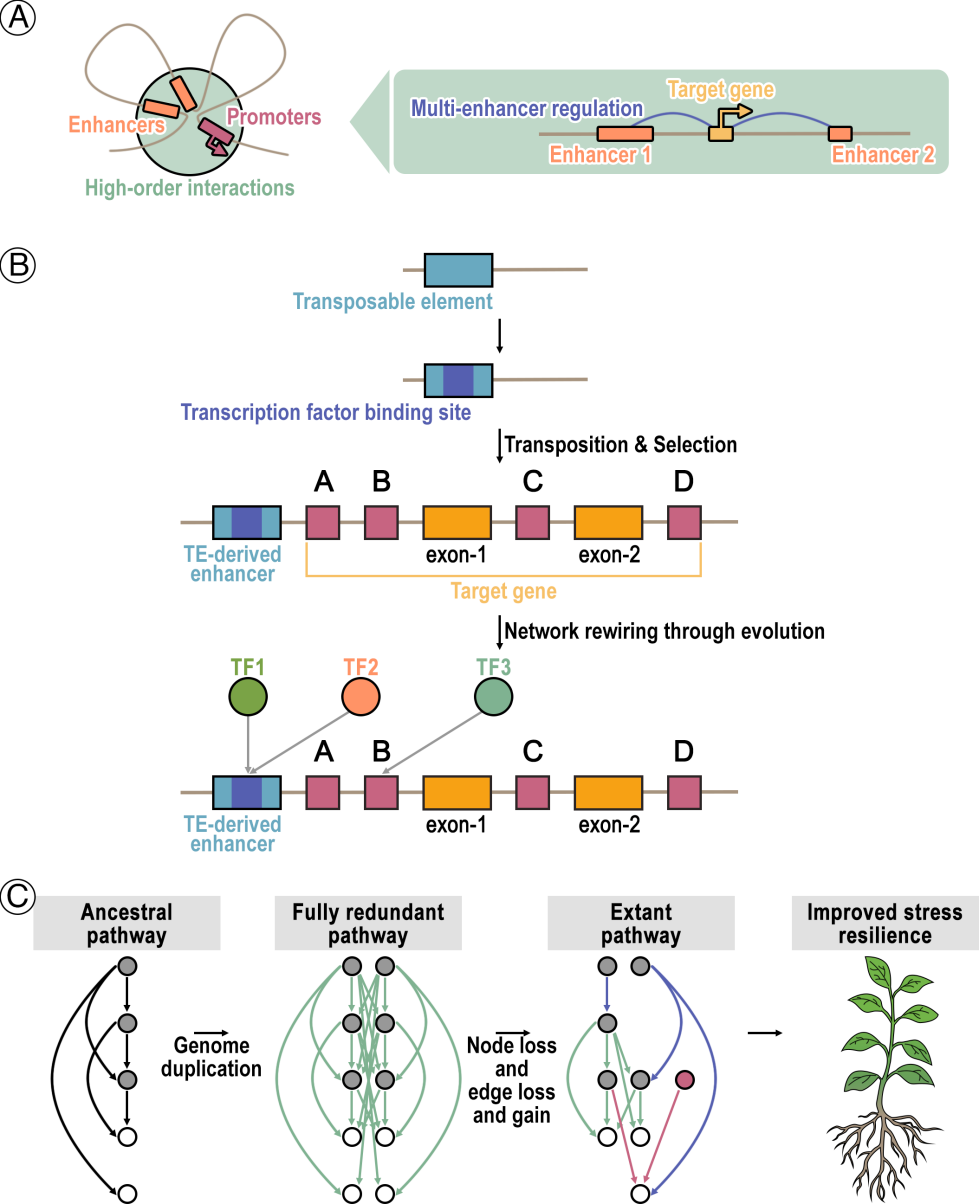
Regulatory evolution and network rewiring in polyploids. Panel (*A*) depicts higher-order interactions among a gene’s CREs (e.g., enhancers and promoters). Panel (*B*) illustrates how a TE acquires a TF binding site. The movement of this TE to a new gene can lead to the recruitment of new TFs (TF1 and TF2), resulting in altered gene expression patterns and, potentially, network rewiring. Panel (*C*) depicts pathway evolution following polyploidization (adapted from ref. 42), which occurs at the broader (sub)network levels (e.g., interconnected stress related pathways). It begins with a fully redundant pathway, as seen in a new autopolyploid. Subsequent gene and CRE loss results in network rewiring. The figure shows redundant interactions (green arrows), subfunctionalization (blue arrows), and novel interactions (red arrows). The red node represents a novel gene that was recruited by acquiring a new CRE. Collectively, these processes contribute to new phenotypes and variation, including improved stress resilience.

CREs are noncoding sequences associated with genes, often located in close vicinity, that play a fundamental role in controlling gene expression (37). These noncoding regions are often bound by TFs and other regulatory proteins, acting as molecular switches that determine when, where, and to what extent a gene is turned on or off (Fig. 2*C*) (38). Key types of CREs include promoters, typically found immediately upstream of a gene, which serve as the primary binding site for RNA polymerase to initiate transcription (39). Enhancers, often located at longer distances from the gene(s) they regulate (either upstream, downstream, or within introns), can significantly modulate gene expression (40). Conversely, silencers function to repress or reduce gene activity (41). Together, the interplay of these CREs orchestrates the precise spatial and temporal patterns of gene expression essential for development, cellular function, and response to various environmental stimuli (Fig. 3*A*).

The identification and functional validation of CREs responsive to stress are being significantly advanced by several cutting-edge molecular techniques. Active CREs are known to be embedded in chromatin that are hypersensitive to various nucleases, referred to as “open chromatin” or accessible chromatin regions (ACRs) (43). ACRs can be identified by several genomic methods based on their hypersensitivity to nucleases, including ATAC-seq (Assay for Transposase-Accessible Chromatin with sequencing, based the Tn5 transposase) (44), DNase-seq (based on DNase I) (45), and MH-seq (Micrococcal Nuclease Hypersensitivity sequencing, based on MNase) (46). These methods enable genome-wide identification of potential regulatory sites that become more or less available for TF binding under stress conditions. The newest technologies now enable generating single cell ATAC-seq data, which can be directly paired with single-cell RNA-seq data to produce detailed, cell-type-specific gene expression profiles (47). This is incredibly powerful, moving far beyond organ-specific data to reveal the precise regulatory dynamics within individual cell types. ChIP-seq (Chromatin Immunoprecipitation sequencing), on the other hand, allows researchers to pinpoint the exact genomic locations where specific TFs or histone modifiers (which mark active or repressed chromatin) bind (48). By performing ChIP-seq under different stress treatments, it is possible to identify CREs where key regulatory proteins associate to modulate gene expression (49). Finally, reporter assays serve as an essential validation step. In these experiments, putative CREs are fused to a reporter gene (like GUS or luciferase) and introduced into plants, allowing the activity of the CRE to be directly measured under various stress stimuli, thereby confirming its functional role in stress-responsive gene regulation (50). Putative CREs can also be deleted or modified using CRISPR/Cas-mediated genome editing to determine their function in vivo (51). Together, these methods provide a toolkit for deciphering the regulatory networks that govern stress response and adaptation.

Complementing these lab-based methods, computational approaches leverage large-scale genomic and transcriptomic datasets to predict and characterize CREs. While comparative genomics approaches can efficiently identify conserved regulatory sequences across species, advanced bioinformatics and systems biology algorithms have been developed to discover lineage or species specific CREs. Based on coexpressed stress-responsive genes, Bayesian graphical models and statistical learning algorithms (e.g., EM and MCMC algorithms) have been powerful tools to search for overrepresented DNA motifs of different TF binding sites (e.g., refs. 52–54). More recent methodology innovations include the inclusion of CRE-gene regulatory networks beyond the coexpression networks to jointly analyze enriched motifs in both enhancers and promoters (e.g., ref. 55), the integration of the ATAC-seq data into motif discoveries (56), the capability of integrating single-cell datasets (57), and the inferences of combinatorial motifs of TF binding modules (58). Notably, machine learning models trained on known CRE data have further transformed the interpretation and enrichment analysis of CREs’ DNA sequence patterns into the era of predictive models and synthetic designs. Beyond traditional machine learning frameworks (e.g., SVM-based algorithms) (59), a variety of deep learning models have been built to predict base-resolution cell-type specific chromatin accessibility and TF bindings. These deep learning models employ different architecture (e.g., CNN, transformer, pretrained foundation models) (60–62) and have demonstrated promising accuracy, including cross-species predictions (63). Deep learning models have also been developed to predict chromatin interactions (64), MPRA signals (65), and single-cell gene expressions (66). In addition to the learned DNA signatures of the regulatory grammar, the predictive nature of these models, including the most recent AI model AlphaGenome (67), enables in silico perturbations of CRE sequences or TF binding sites to dissect the downstream effects on gene expressions. Following the framework of deep learning technologies, new algorithms (68–71) have also been developed to design or engineer synthetic CREs with cross-species functionality. Together, these diverse tools enable the comprehensive discovery of CREs involved in responses to a wide array of abiotic and biotic stresses.

Variation within CREs is a fundamental driver of phenotypic diversity and adaptive evolution in plants (72, 73). At the most granular level, sequence polymorphisms, such as single nucleotide polymorphisms (SNPs) and small insertions or deletions, can directly alter the binding affinity of TFs, thereby modulating gene expression. Beyond these subtle changes, PAV refers to the complete gain or loss of a CRE sequence across different individuals within a species, leading to profound differences in regulatory potential (Figs. 1*A* and 2*C*). Furthermore, larger-scale structural variations, including position variations where CREs are translocated, CNVs that duplicate or delete entire regulatory regions, and chromosomal rearrangements, can drastically alter the context of a CRE, bringing it closer to or further from its target gene, or placing it under novel regulatory influences (74). These diverse forms of variation collectively reshape the regulatory landscape, providing the raw material for differential gene expression and adaptation to environmental challenges. Transposable elements (TEs) can play a significant role in this process by capturing existing CREs and moving them to new locations within the genome (Fig. 3*B*). A translocated CRE can become associated with a new gene, leading to novel gene interactions (18) and, consequently, a rewiring of gene regulatory networks that shapes new phenotypes or contributes to phenotypic variation (Fig. 3*C*). For example, Crassulacean acid metabolism (CAM) photosynthesis in pineapple provides a striking example of adaptive evolution, emerging through the reconfiguration of existing C3 plant pathways. This involved the regulatory neofunctionalization of preexisting genes, notably driven by the movement of CREs originally associated with circadian clock genes to key photosynthesis genes (75). Such positional variation of CREs has also been observed in other species, including *Arabidopsis*, where it was shown to be partly driven by TEs, contributing to novel expression patterns in adaptive genes—particularly those and their associated TF motifs linked to abiotic stress tolerance (76).

Divergence in CREs can often lead to more immediate and pronounced phenotypic effects, particularly in polyploids, compared to changes within protein-coding regions of genes [e.g., cotton (77)]. This is because subtle alterations in CREs can drastically reshape gene expression patterns—affecting timing, location, or magnitude—without necessarily changing the protein itself. However, gene dosage changes within protein-coding genes are also well-established contributors to novel phenotypic diversity (78). For instance, modified relative ratios of parental gene copies of particular regulatory genes, a direct consequence of dosage alterations, have been linked to phenotypic variations, such as the observed differences in flowering time in allotetraploid *Brassica napus* (79). Thus, while CRE divergence offers a swift route to phenotypic novelty, changes in gene dosage of protein that bind to the CREs also play a vital, often complementary, role

Connecting variations within CREs directly to observable plant traits remains a significant challenge, although a major focus of ongoing research. Subtle changes within a promoter can dictate whether a stress-responsive gene is expressed only in specific tissues or more broadly throughout the plant (73). For instance, a SNP in an enhancer might shift the timing of a defense gene’s activation, allowing an individual to mount a faster response to herbivore attack (80). Similarly, the PAV of an entire CRE could lead to the complete lack of expression for a salt-tolerance gene in one accession, while another with an intact CRE exhibits robust expression and enhanced survival in saline soils (81). These examples highlight how the precise spatial and temporal orchestration of gene expression, governed by CRE diversity, underpins observable differences in a plant’s ability to cope with environmental adversities, but connecting this to trait variation remains difficult beyond a few individual genes (73).

Finally, while the evolution of CREs is a major driver of change in duplicate genes and pathways, a complete understanding also encompasses the emergence of *trans*-regulatory differences that arise over time. It is important to note that many such regulatory differences may have already evolved in the diploid progenitors, especially in wide allopolyploids, but here, due to space constraints, we are largely discussing postpolyploid evolution. A key mechanism for this is the action of noncoding RNAs (ncRNAs), which orchestrate gene expression from a distance. Small interfering RNAs (siRNAs) play a particularly pivotal role in plant genomes. These ncRNAs, derived from plant-specific RNA polymerases, are processed through the plant-specific dicer pathway and suppress TEs by orchestrating DNA methylation (RNA directed DNA methylation, RdDM) that inhibits transcription. Changes in their abundance can lead to the liberation of TEs in newly formed polyploids (82). This release of TEs, in turn, can induce widespread genomic changes. Furthermore, the differential expression of siRNAs, alongside TE density differences, between subgenomes in allopolyploids is a significant contributor to subgenome expression dominance, where one parental genome exerts a greater influence on the polyploid’s overall gene expression. In addition to siRNAs, other classes of ncRNAs also contribute to this complex regulatory landscape. MicroRNAs (miRNAs) and long noncoding RNAs (lncRNAs) play important regulatory roles (Fig. 1*E*). They can fine-tune gene expression and translation by targeting specific messenger RNAs for degradation or by influencing chromatin structure (83). This layered regulatory control, involving both cis- and trans-acting factors, contributes to the interplay between different subgenomes and ultimately shapes the unique adaptive potential of polyploid species.

## Leveraging Polyploidy to Study Regulatory Variation: The Outcome of Fractionation

As noted above, after whole-genome duplication, newly formed polyploid genomes will undergo fractionation—a process involving the loss of duplicated sequences, such as genes and CREs, driven by both neutral processes and selection (9). This ongoing genetic erosion significantly shapes the evolution of polyploid genomes, leading to more streamlined “diploidized” chromosome sets over time. The patterns of gene and CRE loss are not random; some duplicates are retained due to dosage balance constraints or the emergence of novel functions (neofunctionalization), while others are lost (11). The functional consequences of this fractionation provide a powerful natural experiment: By comparing polyploids where specific genes or CREs have been retained or lost from different subgenomes, especially in comparison to diploid progenitor genomes or extant relatives of the progenitor genomes, if available, researchers can functionally characterize their roles (74). This comparative approach offers valuable insights into the functions of particular genes and can identify CREs with spatiotemporal-specific expression profiles, including those directly associated with stress resilience.

A common phenomenon in allopolyploids is asymmetric fractionation, where duplicated genes and regulatory elements are lost unevenly from the different parental subgenomes (84). This often leads to subgenome dominance, a state where one subgenome contributes disproportionately more to the overall gene expression and functional capacity of the polyploid (85). The consequences for stress-responsive CREs are significant: The dominant subgenome may preferentially retain key stress-related CREs and their associated genes, while the recessive subgenome experiences greater loss or silencing of these regulatory components. It is important to note that for some allopolyploids, the recessive subgenome has been shown to serve more adaptive functions, under the model that it is under relaxed selection to evolve novel functions (86). Additionally, the dominant subgenome has been observed to undergo higher rates of tandem duplications compared to the recessive subgenome (87).The functions of these tandemly duplicated genes are often skewed toward abiotic and biotic stress functions, including known disease resistance genes, suggesting a specialized role in rapid adaptation to environmental challenges. However, despite the apparent dominance of one subgenome, it is important not to dismiss the potential adaptive role of the recessive subgenome for particular stress responses. This is because its diploid progenitor may have been better adapted to a specific abiotic stress or more resistant to a certain pest.

Following polyploidization, duplicated CRE and their associated homoeologous genes can undergo regulatory subfunctionalization (Fig. 2*C*). This process occurs when the ancestral regulatory functions are partitioned between the duplicated copies, rather than one copy becoming entirely nonfunctional (88). For example, the loss of a specific CRE from one duplicated gene copy can result in its expression being restricted to roots under drought stress, while its homoeologous counterpart, having lost an alternative CRE, is now expressed only in leaves during heat stress. This division of labor in gene regulation allows the polyploid to develop more refined and nuanced responses to diverse environmental challenges. The distinct spatial and temporal expression patterns of these homoeologous stress-related genes, orchestrated by subfunctionalized CREs, contribute significantly to the broader adaptive capacity and resilience often observed in polyploid species. This process provides a unique opportunity to functionally characterize the role of specific CREs in a “natural knockout” experiment by evaluating the expression differences of the two homoeologous genes, especially when compared to a diploid outgroup that still contains the complete ancestral array of CREs.

**Fig. 4.**
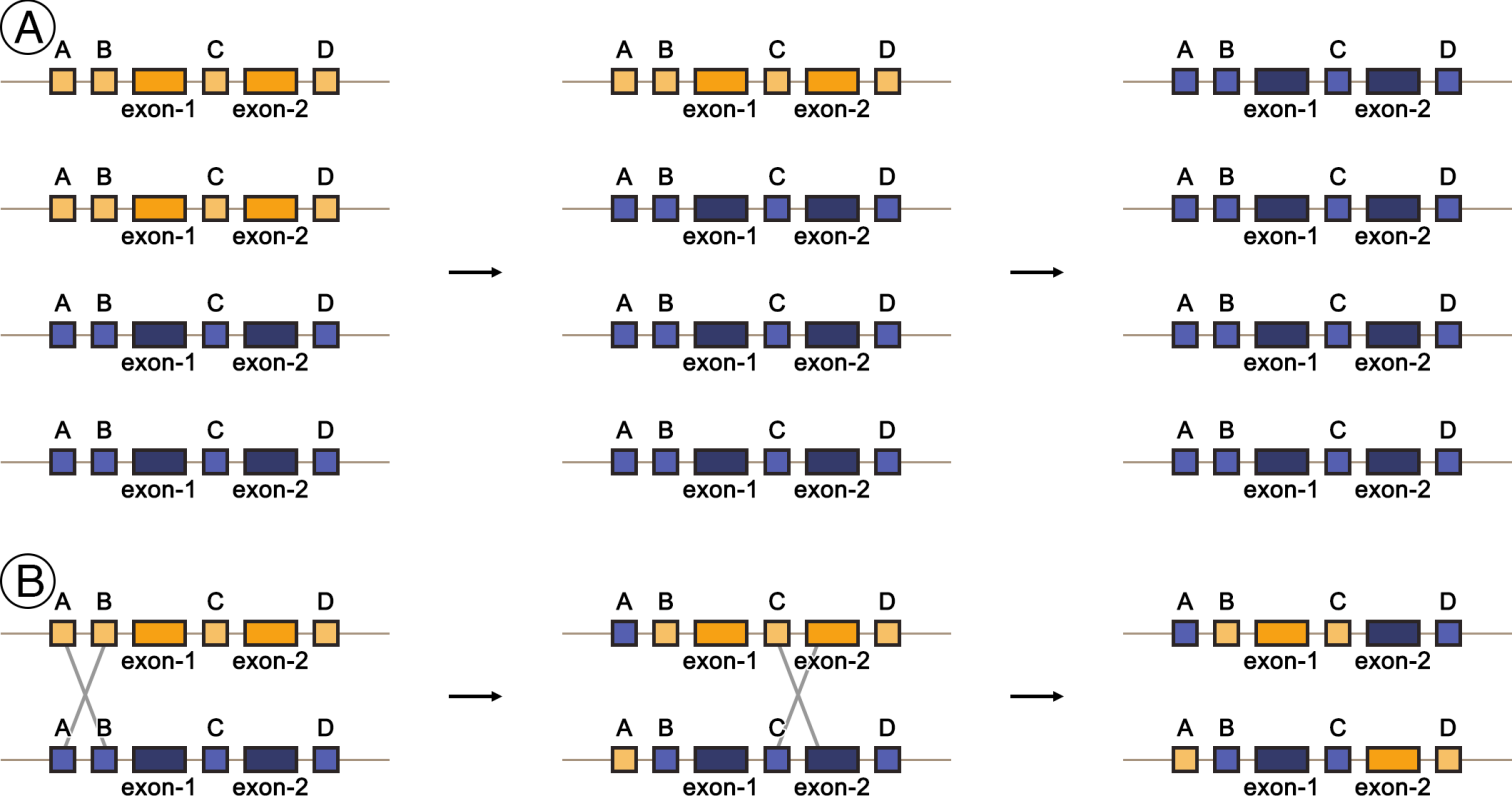
HE and its impact on gene dosage and structure. This figure illustrates how HEs, which are recombination events between chromosomes from different diploid progenitors (shown in blue and yellow), can alter gene dosage and create novel gene structures in a polyploid genome. Panel (*A*) depicts the potential outcomes of HE on homoeolog dosage. A newly formed tetraploid begins with two copies from each parental genome (*Left*). HEs and subsequent segregation can alter this ratio to 3:1 or 1:3 (*Middle*) and, in extreme cases, to 4:0 or 0:4 (*Right*). Panel (*B*) shows that HEs can also occur within a gene. These events can either alter CRE variants associated with a gene (*Left* to *Middle*) or, in more extreme cases, create chimeric versions of genes consisting of exons from both homoeologs (*Middle* to *Right*).

HE—the reciprocal recombination between chromosomes from different progenitor genomes—introduces another dynamic layer to regulatory variation (Fig. 2*B*). HE events can drastically alter the local dosage of parental gene copies and their associated CREs, potentially creating new combinations of CREs associated with parental copies, protein fusion variants or forming chimeric fusions that consist of regulatory elements from both parents (89) (Fig. 4). For example, a HE might lead to the gain of a highly active CRE from one subgenome while simultaneously losing a less effective one from its homoeologous counterpart. This dynamic reshuffling changes the relative contribution of each parental regulatory landscape to the expression of specific genes, thereby modifying their dosage and potentially leading to novel or altered stress response phenotypes. Such changes in regulatory dosage, driven by HEs, offer a rapid evolutionary mechanism for fine-tuning adaptive traits in polyploids under environmental pressures. This results in significant variation in gene and CRE composition among and between polyploid populations, reflecting adaptations to local environments. This is a key reason why polyploids have long been characterized as being highly plastic, at the population level, compared to their diploid progenitors, as they possess additional genetic variation that can be readily selected upon for adaptation and for creating new, superior cultivars (12).

## Exploiting Regulatory and Gene Content Variation for Crop Improvement

Harnessing the power of gene content and regulatory variation in polyploids offers promising avenues for enhancing stress resilience in crop breeding (7). While traditional breeding methods often rely on slow, phenotype-based selection, the identification of beneficial gene and CRE variants opens up exciting opportunities for precision strategies like marker-assisted selection (MAS) and genomic selection (GS) (90). MAS allows breeders to directly select for desirable alleles using molecular markers, accelerating the development of resilient varieties. Similarly, GS, which uses genome-wide marker data to predict breeding value, can incorporate information about favorable variants to identify resilient parents and progeny. Beyond specific gene and CRE variants, these strategies can also focus on selecting gene dosage—both the total number of gene copies and the relative contribution of parental gene copies in allopolyploids—to enhance stress resilience. In polyploids, duplicated genes offer a reservoir of variation, where altering the overall number of active copies of a particular gene or the relative balance between homoeologous copies can significantly impact the strength or duration of a stress response. By strategically breeding for desired gene dosages alongside beneficial regulatory variants, breeders can more efficiently introduce or combine genetic elements that confer robust responses to various abiotic and biotic stresses, ultimately leading to more climate-resilient crops.

The advent of CRISPR-Cas and other advanced genome editing technologies has revolutionized the ability to precisely engineer plant genomes, offering unprecedented control modifying various traits (91). These tools allow for the targeted modification of CREs, enabling researchers to fine-tune gene expression patterns by altering TF binding sites, thereby enhancing or repressing specific pathways (e.g., ref. 92). Beyond CREs, genome editing can also be employed to directly manipulate gene copy numbers, either by introducing additional copies of beneficial genes or by excising redundant or deleterious ones. For example, targeted mutations in the coding region of a single gene in maize may alter water use efficiency, a key trait for improved drought tolerance (93). This precise alteration of gene dosage, coupled with targeted CRE modifications (94), provides a powerful means to optimize a plant’s genetic architecture for improved tolerance to various abiotic and biotic stresses, accelerating the development of next-generation climate-resilient crops.

## Conclusions and Future Perspectives

Polyploidy is now understood to drive significant shifts in gene content and *cis*-regulatory variation, both of which are fundamental to plant stress resilience. Gene content variation, including the gain or loss of specific genes, directly impacts metabolic and signaling pathways vital for adapting to environmental challenges. Simultaneously, changes in CREs lead to diverse and novel patterns of gene activity that fine-tune stress responses. This dynamic interplay between genetic and regulatory changes contributes to the expanded phenotypic diversity and enhanced adaptive capabilities that enable polyploids to thrive under challenging conditions.

A comprehensive understanding of regulatory variation in polyploids holds immense potential for developing next-generation, climate-resilient crops and ensuring global food security. While the added phenotypic diversity, genetic redundancy, and multiple gene variants in polyploids provide a rich substrate for adaptation, they have historically made it difficult to apply molecular breeding approaches. For this reason, some breeding communities have opted to work with diploid progenitors, sacrificing valuable phenotypic diversity for the simplicity and lower cost of genetic markers. The advent of new genotyping and bioinformatic tools, combined with the development of haplotype-phased pangenomes, is making the use of molecular breeding tools with polyploids increasingly more accessible and affordable for a broader range of communities working on a wide variety of polyploid crops.

While significant progress has been made in understanding gene and CRE evolution following polyploidy, many aspects remain poorly understood. It is likely that insights into stress adaptations and resilience are likely to derive from these five key research areas.

1 Dissecting Regulatory Complexes: Understanding which CREs function together to regulate a single gene is a major challenge. Future research could leverage gene and CRE fractionation in polyploids to identify elements that function as a macromolecular complex. By correlating the co-loss of specific CREs from a duplicate gene copy with a loss of function, we can infer physical or functional interactions. This “co-loss,” analysis offers a powerful method to dissect modular gene regulation, which can be complemented with other more traditional methods. Systems-level modeling will further clarify interdependencies among TFs, CREs, and genes forming sophisticated regulatory networks.
2 Mapping the 3D Regulatory Architecture in Polyploids: The larger-scale, three-dimensional architecture of the genome and its influence on gene expression remains poorly understood, especially in polyploids. Future studies might consider focusing on mapping the complete network of long-distance interactions between more distal enhancers and their target genes across different subgenomes. By integrating this 3D chromatin conformation data with single-cell genomics, researchers can uncover how HE events and other structural variations rewire these interactions, leading to novel or altered stress response phenotypes.
3 Integrating CREs with Coexpression Networks to Uncover Regulatory Drivers: Future research could focus on connecting CRE changes to gene regulatory network rewiring. A priority is integrating CRE variation with coexpression networks to identify master regulators and their targets. Mapping altered CREs onto coexpression modules will uncover how they modulate network dynamics under stress, clarifying how DNA-level regulatory shifts translate into coordinated gene expression and phenotypic variation.
4 TE-Driven Regulatory Evolution and Network Rewiring: TEs are increasingly recognized for shaping polyploid regulatory evolution by contributing to novel gene expression and network rewiring. However, the overall frequency and evolutionary impact of these events, particularly how often stress-adaptive traits arise from novel TE insertions moving CREs to new genes, remain poorly understood.
5 Predicting Phenotypes from Pangenome-Level Variation: Leveraging pangenome diversity for crop improvement requires better phenotypic prediction. While we can catalog gene content and CRE variation, accurately linking specific accessory genes and/or CRE variation to superior stress resilience is limited. Developing predictive models could help with translating genomic data into actionable breeding strategies.

## Data Availability

There are no data underlying this work.
